# Magnetic resonance imaging in the diagnosis of HTLV-1-associated myelopathy

**DOI:** 10.1055/s-0045-1809935

**Published:** 2025-07-22

**Authors:** João Marcos Carvalho, Sheila Nunes Ferraz, José Abraão Neto, Saul Schnitman, Augusto M. Carvalho, Edgar M. Carvalho

**Affiliations:** 1 Universidade Federal da Bahia, Hospital Universitário Professor Edgard Santos, Serviço de Imunologia, Salvador BA, Brazil.; 2 Universidade Federal da Bahia, Programa de Pós-Graduação em Ciências da Saúde, Salvador BA, Brazil.; 3 Universidade Federal da Bahia, Hospital Universitário Professor Edgard Santos, Serviço de Neurologia, Salvador BA, Brazil.; 4 Instituto Nacional de Ciência e Tecnologia de Doenças Tropicais (INCT-DT), Salvador BA, Brazil.; 5 Fundação Oswaldo Cruz, Instituto Gonçalo Moniz, Salvador BA, Brazil.

**Keywords:** Human T-lymphotropic Virus 1, Paraparesis, Tropical Spastic, Magnetic Resonance Imaging, Lower Urinary Tract Symptoms

## Abstract

**Background:**

The main neurologic manifestation of definitive human T-lymphotropic virus 1 (HTLV-1)-associated myelopathy (HAM) is spastic paraparesis, but it only occurs in 5% of the patients. In contrast, about 40% of HTLV-1-infected subjects present symptoms of urologic dysfunction, including nocturia, urgency, and incontinence, which may progress to an inability to void urine. As these patients do not present motor dysfunction, they are classified as *probable HAM* . Atrophy of the thoracic spinal cord (SC) is the main abnormality found on magnetic resonance image (MRI) scans of patients with definitive HAM, but damage to the SC has not been reported in patients with probable HAM.

**Objective:**

To determine if, through an evaluation of the metrics of conventional MRI, we can detect a decrease in the area of the SC in patients with probable HAM.

**Methods:**

Infection by HTLV-1 was herein diagnosed by a Western blot, and the MRI scan was performed using a 1.5-T scanner. Atrophy was considered when the SC area was less than 25% of the intrathecal area.

**Results:**

We observed a progressive reduction in all segments of the SC area among HTLV-1 carriers, patients with probable and definitive HAM. Significantly, 48.3% of patients with probable HAM presented atrophy of the lumbar area.

**Conclusion:**

Using MRI metrics, the present study shows the atrophy of lumbar segments of the SC area in patients who present urinary symptoms associated with HTLV-1 but without motor dysfunction.

## INTRODUCTION

Human T-lymphotropic virus type 1 (HTLV-1) was the first human retrovirus identified. 1 It is estimated that 5 to 10 million people are infected with HTLV-1 worldwide, with a high prevalence in the tropical areas of developing countries. 2 3 The two major diseases associated with HTLV-1 infection are adult T-cell leukemia/lymphoma and HTLV-1-associated myelopathy (HAM). 4 However, HTLV-1 infection is associated with various other diseases or clinical manifestations, such as urinary and erectile dysfunctions, sicca syndrome, infective dermatitis, and arthropathy. 5 6 7 8 9 Patients who present spastic paraparesis are classified as *definitive HAM* . Those who present urinary, sexual, and/or intestinal dysfunctions or monosymptomatic neurologic findings, but do not present motor disability, are classified as *probable HAM* . Meanwhile, most of the infected individuals are HTLV-1 carriers. 10 Neurological manifestations in patients with definitive HAM include lumbar pain, hyperreflexia, the Babinsky sign, and a progressive spastic paraparesis leading to an inability to walk. 10 Patients with probable HAM primarily manifest urinary dysfunction. 11 12 They complain of nocturia, urgency, incontinence, and later develop areflexic bladder requiring catheterization to void urine. 5 13

The diagnosis of definitive HAM is primarily based on clinical and neurological manifestations. Spinal cord (SC) atrophy observed through inspection of magnetic resonance imaging (MRI) scans presents low sensitivity. 14 15 16 Diffusion tensor imaging (DTI) improves SC assessment compared to conventional MRI by detecting microstructural alterations in the white matter before the atrophy becomes visible. Its quantitative metrics, such as fractional anisotropy (FA) and mean diffusivity (MD), enable the early identification of axonal damage and demyelination, increasing diagnostic sensitivity. Additionally, DTI-based tractography enables the visualization of spinal tracts, aiding in the identification of specific impairments. Moreover, DTI offers a complementary analysis to conventional MRI by providing insight into the integrity of white-matter fibers. While conventional MRI reveals morphological changes such as SC atrophy, DTI is more sensitive to microstructural alterations, such as axonal loss and demyelination, often detecting these changes before they are visible on structural images. This ability to detect early-stage damage enhances diagnostic sensitivity, which is particularly important in conditions such as HAM, in which clinical symptoms and atrophy may emerge later in the disease course. 17 18 Alternatively, the use of automated and semiautomated tools, such as the SC Toolbox (SCT; open source) software, NeuroQlab (Fraunhofer Institute for Digital Medicine MEVIS) software, and 3D Slicer (free and open source) software, enable a more reproducible and faster segmentation, reducing observer bias and increasing measurement accuracy. However, these approaches rely on specific software and trained neuroradiologists, and often require images acquired with 3-T MRI scanners, which are not widely available to a large portion of the population. This limits their applicability in the clinical practice and in resource-limited centers.

Validating an efficient technique to measure the SC area that does not require specific software or high-cost equipment would be extremely useful, making the analysis more accessible and facilitating its incorporation into the routine clinical practice. In the present study, we have used a method to measure the cross-sectional area of the SC through the conventional MRI scanner. 14 We opted for manual SC area measurement, as it is feasible using images acquired with a 1.5-T MRI scanner, making it more applicable in diverse clinical settings. Urinary manifestations (mainly overactive, but also areflexic bladder) are observed in high frequency in HTLV-1-infected individuals and reduce quality of life. 19 However, an important gap in this field is the lack of studies aimed at determining whether individuals with urinary dysfunction but no motor disability also present SC damage. The present study aimed to determine if, through an evaluation of the conventional MRI metrics, we could detect a reduction in the SC area not only in patients with definitive HAM but also in those who have probable HAM.

## METHODS

The current is a cross-sectional study with the participation of 101 HTLV-1-infected subjects, including 11 seronegative controls, at the Multidisciplinary HTLV-1 Clinic of Hospital Universitário Professor Edgard Santos, located in the city of Salvador, Brazil. The HTLV-1-infected subjects were aged 18 years or older, of both genders, and they presented positive serology for HTLV-1 by enzyme-linked immunosorbent assay (ELISA; Cambridge BiotechCorp.), confirmed by Western blot (HTLV blot 2.4; Genelab). The exclusion criteria were patients diagnosed with myelopathy due to other causes, positive serology for HIV or HTLV-2, and inability to undergo MRI.

All subjects filled out a standardized questionnaire and underwent neurological physical examinations. The proviral load was also quantitatively determined. Three scales were used to assess neurological disfunction in individuals infected with HTLV-1: the Overactive Bladder Symptom Score (OABSS), 20 the Osame Motor Disability Score (OMDS), 21 and the Extended Disability Status Scale (EDSS). 22 Patients with definitive HAM presented spastic paraparesis and an OMDS greater than 1. Patients classified as *probable HAM* were HTLV-1-infected subjects who presented urinary symptoms of overactive bladder, such as urgency, incontinence, nocturia, and/or polyuria, with an OMDS of 0.

Conventional MRI was performed with a 1.5-T scanner (Siemens), using a 16-chanel spinal coil. The protocol consisted of a sagittal T2-weighted images (turbo spin echo sequence echo time/repetition time [TE/TR] = 131/5,010 ms; 3.0-mm slice thickness, filed of view [FOV] = 320 mm), three-dimensional sampling perfection with application-optimized contrasts using different flip angle evolution (SPACE 3D) sequence (TE/TR = 124/1,500 ms; measured voxel size = 1.0 × 1.0 × 1.0mm; FOV = 256 mm) and transversal T2-weighted sequence (TE/TR = 95/4,990 ms; FOV = 170mm). The evaluation of the spinal cord was done using T2-weighted images. The T2-weighted images are effective for visualizing the morphology of the spinal cord, enabling the identification of distortions, lesions, and atrophy; in addition, they can detect pathological changes such as inflammatory lesions, demyelination, and edema with good clarity.

**Figure 1:**
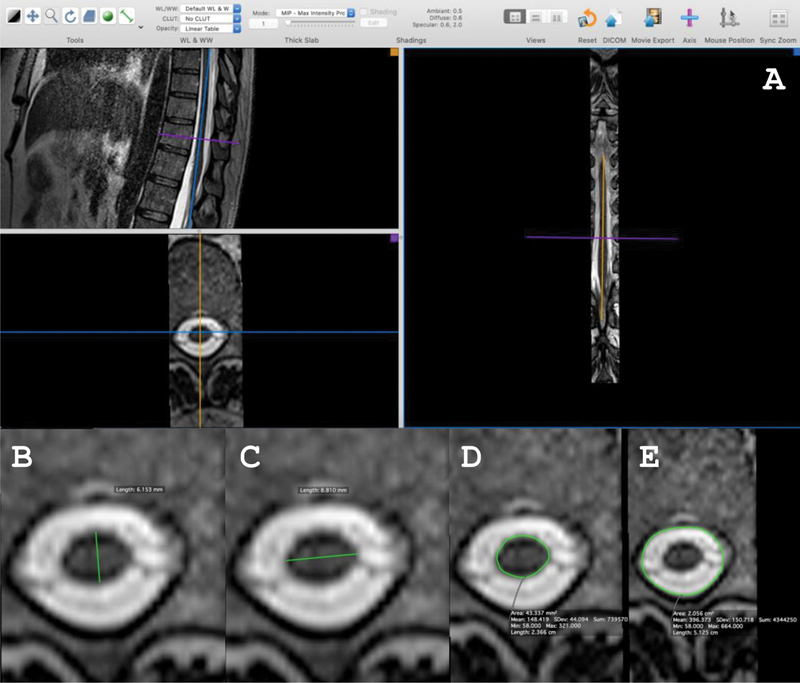
Multiplanar analysis of the lumbar segment of the spinal cord with its metrics at the level of the D11 vertebral body in a patient with human T-lymphotropic virus 1 (HTLV-1)-associated myelopathy (HAM). ( **A** ) Multiplanar image; ( **B** ) Anteroposterior diameter measurement; ( **C** ) Transversal or latero-lateral diameter measurement; ( **D** ) Spinal cord cross-sectional area measurement; and ( **E** ) Intrathecal area measurement.

In the present study, we chose to analyze the images exclusively in T2 to reduce the time and costs involved in the evaluation process. Thus, we aimed to make MRI analysis more accessible without compromising the identification of key SC changes, such as atrophy and lesions, making the approach more feasible for centers with limited resources. The diameters of the cervical, thoracic, and lumbar SC segments cord were measured at the levels of the vertebral bodies of C3, D7, and immediately above the conus medullaris respectively, using a multiplanar analysis technique (sagittal, coronal, and axial) via the Horos software (free and open source), version 4.0.1, with manual measurements of the image of the evaluated spinal segment in the true axial plane ( Figure 1 ), obtained by correcting the image in the other planes (sagittal and coronal). The anteroposterior diameter was defined as the distance from the ventral to the dorsal borders, while the transversal diameter was the distance from the left to the right borders of the SC. The transverse area of the SC was defined as the area of the cross-section delineated by the SC outer border, excluding the cerebrospinal fluid (CSF) and other surrounding structures. The intrathecal area was obtained by measuring the section that encompassed the subarachnoid space, which includes both the SC and the CSF surrounding it. The intrathecal area encompasses the entire region around the SC, from the ventral to the dorsal borders, including the CSF zone. The atrophy index was calculated as follows: (SC area\intrathecal area) ×100. The SC segment was considered atrophic when the atrophic index was lower than 25%. 14 The analysis was performed independently by two radiologists, and, in case of disagreement, a third radiologist was consulted. The radiologists were blinded regarding the clinical information and final diagnosis.

The current study was approved by the Ethics and Research Committee of the School of Medicine of Universidade Federal da Bahia (CAAE: 13518813.5.0000.5577). All participants signed an informed consent form.

### Statistical analysis

The categorical data were compared using the Fisher's exact test. Comparisons of continuous variables between 2 groups were performed through the Mann-Whitney test, and those involving 3 or more groups, through the Kruskal-Wallis test followed by Dunn multiple comparison tests. Data are expressed as median and interquartile range (IQR) values. The analyses were conducted using the IBM SPSS Statistics for Windows (IBM Corp.) software, version 22.0, and differences were considered significant when *p* < 0.05.

## RESULTS

The demographic and clinical features of the HTLV-1-infected patients and the control group are presented in Table 1 . There was no difference in age, but there was a predominance of women among the patients with definitive and probable HAM. The illness duration was similar between patients with definitive and probable HAM, and there was no difference in the follow-up time. The EDSS and OMDS scores were higher in patients with definitive HAM than in those with probable HAM ( *p* < 0.05), but the OABSS score was similar in those 2 groups ( *p* > 0.05). Of the 31 patients with probable HAM, 14 (48.4%) presented voiding dysfunction due to areflexic bladder and required clean intermittent catheterization (CIC) of the urethra. The MRI data from the patients in the four groups are shown on Table 2 . Atrophy of the SC by inspection (subjective atrophy) was observed in all 3 groups of HTLV-1 infected subjects, but it was predominantly observed in those with definitive HAM. Atrophy was identified based on the atrophy index. However, as the intrathecal area of the spinal canal varies among individuals and atrophy was considered when the SC was less than 25% of the intrathecal area, we compared the spinal canal among the groups. There was no difference in the spinal canal among the groups in the cervical and thoracic segments ( *p* > 0.05). In the lumbar region, there was a difference when the groups with HAM were compared with the seronegative controls ( *p* = 0.03). However, the data in the 3 metagroups (HAM, probable HAM, and HTLV-1 carriers) were comparable ( *p* = 0.1). The use of metrics increased the sensitivity in detecting SC atrophy by 1.9-, 2.5-, and 4.0-fold in patients with definitive HAM, probable HAM, and HTLV-1 carriers respectively compared to the seronegative controls. In patients with definitive HAM and in subjects with probable HAM and HTLV-1 carriers, the SC area in the cervical, thoracic, and lumbar regions was lower than in the seronegative controls. There were no differences in any of the three segments between patients with probable and definitive HAM, as well as between subjects with probable HAM and HTLV-1 carriers, except in the lumbar region of the patients probable HAM compared to HTLV-1 carriers ( *p* ≤ 0.02).

**Table 1:** Demographic data, clinical features, and proviral load of the study sample

Characteristics	Definite-HAM patients (n = 26)	Probable-HAM patients (n = 31)	HTLV-1 carriers (n = 44)	Seronegative controls (n = 11)	*p* -value
Mean age (years)	52.5 ± 11.8	59 ± 10.7	55.5 ± 16.2	60 ± 14.4	0.05 ^a^
Female sex: n (%)	19 (73.1)	23 (74.2)	19 (43.2)	6 (54.5)	0.04 ^b^
Mean follow-up (years)	5.5 ± 4.9	12 ± 5.6	11 ± 5	—	0.06 ^a^
Mean time of illness onset (years)	4.5 ± 4.5	4 ± 2.8	—	—	0.14 ^c^
Mean EDSS score	5.5 ± 1.34	1 ± 1.7	0	—	< 0.01 ^a^
Mean OMDS score	5 ± 2.6	0	0	—	< 0.01 ^a^
Mean OABSS score	7 ± 4.8	8 ± 3.9	1 ± 0.6	—	< 0.01 ^a^
Diabetes: n (%)	6 (23.1)	5 (16.1)	4 (9.3)	0	0.19 ^b^
Hypothyroidism: n (%)	0	1 (3.2)	2 (4.7)	0	0.65 ^b^
HBV infection: n (%)	0	1 (3.2)	1 (2.3)	0	0.77 ^b^
HCV infection: n (%)	1 (3.8)	1 (3.2)	2 (4.7)	0	0.90 ^b^
Mean proviral load (copies/10 ^6^ PBMC)	93,893 ± 198,653.9	32,767 ± 80,857.6	19,745 ± 123,272.9	—	0.12 ^a^

Abbreviations: HAM, human T-lymphotropic virus1-associated myelopathy; HBV, Hepatitis B virus; HCV, Hepatitis C virus; HTLV-1, human T-lymphotropic virus 1; EDSS, Extended Disability Status Scale; OMDS, Osame Motor Disability Score; OABSS, Overactive Bladder Symptom Score, PBMC, Peripheral Blood Mononuclear Cells.

Notes: ^a^ Kruskal-Wallis test; ^b^ Fisher's exact test; ^c^ Mann-Whitney U Test.

**Table 2:** Magnetic resonance imaging data of the study sample

Characteristics		Definite-HAM patients (n =2 6)	Probable-HAM patients (n = 31)	HTLV-1 carriers (n = 44)	Seronegative controls (n = 11)	*p* -value
Subjective atrophy ^c^ present: n (%)		12 (46.2)	4 (12.9)	2 (4.5)	0	< 0.01 ^a^
Objective atrophy ^d^ present: n (%)		18 (69.2)	10 (32.3)	8 (18.2)	0	< 0.01 ^a^
Cervical cord: mean (interquartile range)	Area (cm ^2^ )	0.634 (0.332–0.761)	0.680 (0.418–0.969)	0.703 (0.537–1.040)	0.779 (0.673–1.032)	< 0.01; ^b^ < 0.01;* <0.01; ^¥^ 0.02 ^&^
	AP (cm)	0.680 (0.536–0.806)	0.706 (0.498–0.867)	0.730 (0.617–0.872)	0.784 (0.652–0.913)	< 0.01; ^b^ 0.04;* < 0.01; ^¥^
	LL (cm)	1.196 (0.848–1.343)	1.165 (1.037–1.529)	1.225 (1.044–1.552)	1.271 (1.162–1.424)	0.01; ^b^ < 0.01 ^¥^
Thoracic cord: mean (interquartile range)	Area (cm ^2^ )	0.316 (0.181–0.608)	0.350 (0.255–0.542)	0.392 (0.264–0.718)	0.466 (0.350–0.673)	< 0.01; ^b^ < 0.01;* < 0.01; ^¥^ & *p* < 0.01 ^&^
	AP (cm)	0.537 (0.381–0.732)	0.568 (0.509–0.763)	0.603 (0.486–0.823)	0.654 (0.525–0.720)	< 0.01; ^b^ < 0.01;* < 0.01 ^¥^
	LL (cm)	0.699 (0.552–1.043)	0.768 (0.604–0.944)	0.807 (0.654–1.053)	0.887 (0.772–1.108)	< 0.01; ^b^ 0.01;* < 0.01; ^¥^ 0.01 ^&^
Lumbar cord: mean (interquartile range)	Area (cm ^2^ )	0.393 (0.224–0.668)	0,417 (0.299–0.696)	0.495 (0.364–0.665)	0.630 (0.438–0.811)	< 0.01; ^b^ 0.01;* < 0.01; ^¥^ < 0.01 ^&^
	AP (cm)	0.617 (0.468–0.850)	0.629 (0.566–0.858)	0.675 (0.576–0.828)	0.758 (0.690–0.894)	< 0.01; ^b^ 0.03;* < 0.01; ^¥^ < 0.01 ^&^
	LL (cm)	0.808 (0.597–0.977)	0.820 (0.667–1.029)	0.865 (0.694–1.029)	0.909 (0.854–1.029)	< 0.01; ^b^ < 0.01; ^¥^ < 0.01 ^&^

Abbreviations: AP, anteroposterior; LL, Latero-lateral; HAM, human T-lymphotropic virus1-associated myelopathy; HTLV-1, human T-lymphotropic virus 1; Abbreviation: HAM, HTLV-1-associated myelopathy.

Notes: ^a^ Fisher's exact test; ^b^ Kruskal-Wallis test; ^c^ visual analysis by the radiologist; ^d^ objective atrophy: cross-sectional spinal cord area less than 25% to the intrathecal area; *level of significance for the difference between definite-HAM patients and. HTLV-1 carriers; ^¥^ level of significance for the difference between definite-HAM patients and seronegative controls (Dunn's test); and ^&^ level of significance for the difference between probable-HAM patients and seronegative controls (Dunn's test).

The SC area of the lumbar segment in patients with probable HAM was quite variable. In 15 of them, the area was ≤ 0.417 cm ^2^ , which represents the median SC area in the lumbar segment in subjects with probable HAM. Notably, patients with probable HAM with areas ≤ 0.417 cm ^2^ presented a greater SC area in the lumbar segment than patients with definitive HAM ( *p* = 0.006). We also observed that while 11 (78.5%) of the 15 patients with probable HAM who had a lumbar SC area > 0.417 cm ^2^ required CIC, while only 4 (25%) of the 16 patients with areas > 0.417 cm ^2^ required CIC ( *p* = 0.006).

## DISCUSSION

Atrophy of the thoracic segment of the SC is the main finding in HTLV-1-infected subjects presenting severe illness with motor dysfunction and diagnosed as *definitive HAM* . 14 23 24 In the current study, SC atrophy was observed not only in patients with definitive HAM, but also in those with probable HAM and even, to some extent, in HTLV-1 carriers.

The predominance of women among patients with definite and probable HAM is consistent with previous studies. 2 3 5 The older age in patients with definite and probable HAM compared to HTLV-1 carriers supports the notion that progression to more severe clinical manifestations is associated with aging. 25 26 The higher frequency of HAM in women may be related to immunological differences, including a more intense inflammatory response to HTLV-1, potentially exacerbating SC damage. Additionally, studies suggest that the proviral load tends to be higher in women, a factor associated with disease progression. Sociocultural factors may also contribute, as women generally seek medical care more frequently, increasing diagnosis rates. However, the difference in gender proportion among the groups does not appear to have impacted the study's findings, as no significant differences were observed in terms of age, duration of the infection, or proviral load that would justify a gender-related bias in the results. Future studies with larger samples may further explore the influence of sex on HAM progression and neuroimaging findings.

In the clinical setting, MRI analysis of the SC is typically performed by inspection, characterized as a subjective evaluation by the radiologist. However, when image metrics are taken into account, the technique's sensitivity to detect SC atrophy increases. 18 27 In the present study, we have shown that, in addition to the thoracic segment, the SC areas were also reduced in the cervical and lumbar segments, with a progressive reduction in the anteroposterior and lateral diameters in the following order: HTLV-1 carrier, probable HAM, and definitive HAM. These data show that MRI-based SC metrics are strongly associated with the severity of the neurological symptoms and can be a useful indicator of disease progression.

The detection of the decrease in SC diameters in patients with definitive HAM was expected. However, we have shown that a reduction in the SC area may also be observed in patients with probable HAM who present urinary dysfunction but not motor disability, and even in HTLV-1 carriers. In a previous study, 28 we found that abnormalities in the white matter lesion in the brain MRI were similarly observed in patients with definitive HAM and HTLV-1 carriers. In such cases, cognitive function was lower in definitive HAM compared to the carriers, suggesting that evidence of central nervous system (CNS) damage may precede the onset of the neurologic manifestations.

**Figure 2:**
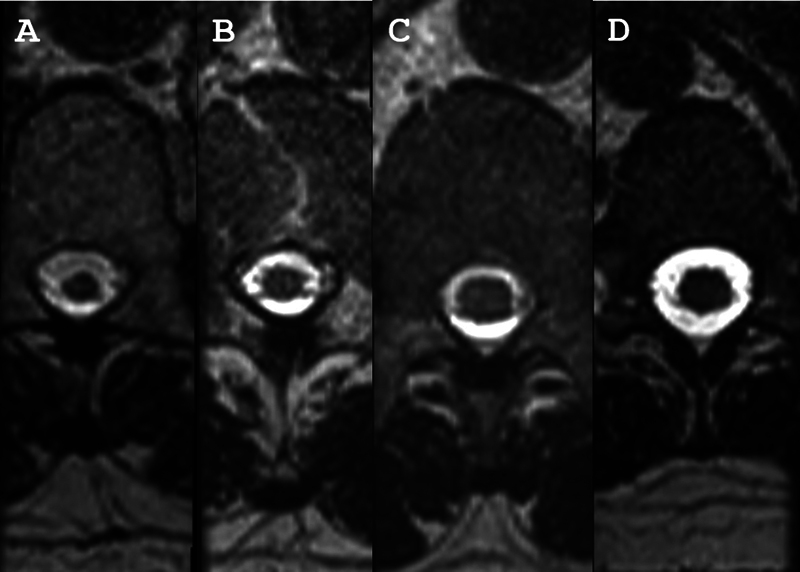
Magnetic resonance imaging scan of the lumbar spine in the axial plane of individuals with definite HAM ( **A** ), probable HAM ( **B** ), human T-lymphotropic virus 1 (HTLV-1) carriers ( **C** ), and seronegative controls ( **D** ).

Urinary dysfunction in HTLV-1-infected subjects is a relevant manifestation not only due to its high frequency, but also due to the decrease in quality of life. 19 While nocturia, urgency, and incontinence are the more common urinary manifestations, a small percentage of these patients present an areflexic bladder and require CIC to void urine. 5 29 Nonetheless, the MRI metrics of the SC in patients with probable HAM have not yet been studied. In the current study, we found that patients with definitive HAM presented a smaller SC area compared to those with probable HAM in the cervical, thoracic, and lumbar segments. However, in a large percentage of the patients with probable HAM, the SC area in the lumbar segment was as small as in patients with definitive HAM. Moreover, a decrease in the lumbar SC area was associated with the severity of the urinary dysfunction ( Figure 2 ).

The current study has some limitations, since the visual assessment was used and semiautomatic or automatic analyses were not performed. To decrease the variability among observers, the analysis was carried out independently by two radiologists. We have also used only the MRI metrics, without the diffusion imaging parameters, as performed by other authors. 17 18 30 Moreover, the 1.5-T MRI scanner with a 16-channel spinal coil provides images with inferior quality compared to 3.0-T scanners. However, our data provides relevant advances and highlights clinical implications regarding the use of the MRI in the HTLV-1 infection. The high prevalence of SC atrophy observed in patients with definitive HAM indicates the need for routine use the metrics of the MRI to monitor disease progression. We also detected atrophy in patients with probable HAM and in HTLV-1 carriers. Additionally, early detection of SC atrophy may enable earlier therapeutic interventions, potentially slowing or preventing disease progression and improving patient outcomes.

The present study shows that patients with urinary dysfunction without motor disability (probable HAM) present atrophy of the lumbar segment of the SC. Moreover, it highlights the use of MRI metrics in detecting SC atrophy in HTLV-1-infected subjects and in monitoring disease progression.
